# Epigenetic Modification of Gene Expression in Honey Bees by Heterospecific Gland Secretions

**DOI:** 10.1371/journal.pone.0043727

**Published:** 2012-08-21

**Authors:** Yuan Yuan Shi, Xiao Bo Wu, Zachary Y. Huang, Zi Long Wang, Wei Yu Yan, Zhi Jiang Zeng

**Affiliations:** 1 Honeybee Research Institute, Jiangxi Agricultural University, Nanchang, Jiangxi, China; 2 Department of Entomology, Michigan State University, East Lansing, Michigan, United States of America; 3 Ecology, Evolutionary Biology and Behavior Program, Michigan State University, East Lansing, Michigan, United States of America; Institute of Genetics and Biophysics, Italy

## Abstract

**Background:**

In the honey bee (*Apis mellifera*), queen and workers have different behavior and reproductive capacity despite possessing the same genome. The primary substance that leads to this differentiation is royal jelly (RJ), which contains a range of proteins, amino acids, vitamins and nucleic acids. MicroRNA (miRNA) has been found to play an important role in regulating the expression of protein-coding genes and cell biology. In this study, we characterized the miRNAs in RJ from two honey bee sister species and determined their possible effect on transcriptome in one species.

**Methodology/Principal Findings:**

We sequenced the miRNAs in RJ either from *A. mellifera* (RJM) or *A. cerana* (RJC). We then determined the global transcriptomes of adult *A. mellifera* developed from larvae fed either with RJM (mRJM) or RJC (mRJC). Finally we analyzed the target genes of those miRNA that are species specific or differentially expressed in the two honey bee species. We show that there were differences in miRNA between RJM and RJC, and that transcriptomes of adult *A. mellifera* were affected by the two types of RJ. A high proportion (23.3%) of the affected genes were target genes of differential miRNAs.

**Conclusion:**

We show for the first time that there are differences in miRNAs in RJ between *A. mellifera* and *A. cerana*. Further, the differences in transcriptomes of bees reared from these two RJs might be related to miRNA differences of the two species. This study provides the first evidence that heterospecific royal jelly can modify gene expression in honey bees through an epigenetic mechanism.

## Introduction

The Western honey bee (*Apis mellifera*) is one of the most important economical insects because of its crucial role in pollination [1]. A honey bee colony is composed of three castes, a fertile queen, hundreds of haploid drones, and thousands of nearly sterile workers [2], [3]. Despite their identical genome, the queen and her workers exhibit vast differences in morphology, behavior, physiology, reproduction and longevity [4]–[6]. The primary substance that leads to this is royal jelly (RJ), which is a yellow milky substance from “nurses” with developed hypopharyngeal and mandibular glands [7]–[9].

RJ contains a range of proteins, carbohydrates, lipids, minerals, vitamins, and a large number of bioactive substances, especially immunological peptides and antibacterial proteins [10]–[13]. Major Royal Jelly Proteins (MRJPs) are the prime RJ ingredient, which are crucial in regulating reproductive maturation [11]. Royalactin is a 57-kDa protein, which can induce larvae developing into queens [14]. Royalactin helps increase body size, promote ovary development and shorten the developmental time. In addition, RJ also contains small amounts of nucleic acids. One study found that RJ contains both DNA and RNA, and there are quantitative differences in nucleic acids in fresh RJ between *A. mellifera* and *A. cerana* [13]. The most recent discovery is that RJ contains microRNAs which may play a role in caste differentiation [15].

MicroRNAs (miRNAs) are short (20–22 nucleotides), non-coding, single-stranded RNA molecules that play important roles in post-transcriptional gene regulation and other biological processes in eukaryotes [16]–[18]. These include development, metabolism and regulation of differentiation [19], [20]. miRNAs may specifically bind to partially complementary sites of targeted genes and inhibit mRNA transcription [21], [22]. Animal miRNAs typically bind to targeted mRNAs with sub-optimal complementarity and inhibit or diminish their translation, whereas plant miRNAs bind with high complementarity and mark them for degradation [17]. miRNAs are first characterized in *C. elegans*, fruit fly (*Drosophila*), honey bee (*A. mellifera*), and mosquito [23]–[28]. miRNAs in the brain are found to correlate with age-related behavioral changes in the honey bee [27], [29]. Young workers specialize on feeding larvae (“nurses”) while workers older than 3 weeks old forage for nectar and pollen (“foragers”) [30]. Nurses and foragers have 9 known differentially expressed miRNAs and 67 novel miRNAs [28]. Two recent studies identified 267 novel miRNAs in *A. mellifera* [31], [32].

While the mechanism by which miRNA modulate gene expression has been well studied [17], [21], [23], it is not clear whether there are differences in RJ originated miRNAs between two honey bee species (*A. mellifera* and *A. cerana*), and whether feeding *A. mellifera* with different RJ might cause differences in transcriptome. *A. cerana* is considered to the most closely related species to *A.mellifera* [33]. The two species share common features (such as open nesting) [34] but show differences in behaviors and physiology [35]. It is likely that the two species diverged from a common ancestor around three million years ago [36]. In this study we report the differences in miRNA of RJ either from *A. mellifera* (RJM) or *A. cerana* (RJC). We also tested the hypothesis that differentially expressed miRNAs in the RJ of the two species affect *A. mellifera* transcriptome.

## Results

### 1. Differences in miRNA between Royal Jelly of Two Different Species of Honey Bees

**Figure 1:**
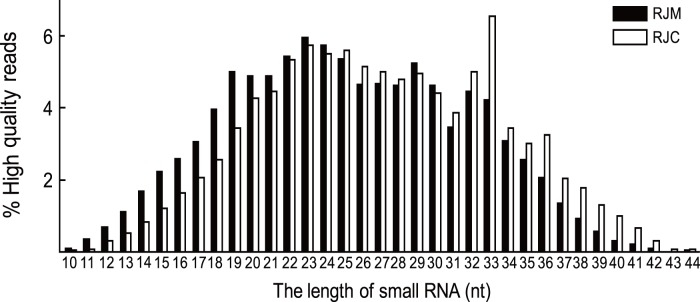
Length distribution of tags produced by small RNA sequencing in Royal jelly of *Apis mellifera* (RJM) and Royal jelly of *Apis cerana* (RJC). The horizontal axis indicated length nucleic acid (nucleotides, Nt), the ordinate represented distribution frequency (%).

A total of 11,828,863 reads from the RJM library and 10,289,838 reads from the RJC library were obtained after discarding the empty adapters (Table 1). After discarding those sequences that were of low-quality, shorter than 18 nucleotides and single-read sequences, 10,318,386 and 9,493,118 reads for the RJM and RJC remained for analysis respectively. RNAs sequenced by Solexa were in the length of 10–44 nucleotides (nt), and length distributions of small RNAs in the two libraries were significantly different (Contingency Table Analysis, X^2^>1000, P<0.001, Fig. 1). miRNAs, those with 20–22 nt, in the two types of RJ also showed significantly different length distributions (Contingency Table Analysis, X^2^>1000, P<0.001).

**Table 1:** Summary of data cleaning and length distribution of tags produced by small RNA sequencing.

	Total reads	High quality (%)	3’adapter null (%)	Insert null (%)	5’adapter contaminants (%)	<18 nt (%)	PolyA (%)	Clean reads (%)
RJM	11,828,863	11,787,392 (100)	58,528 (0.50)	2,191 (0.02)	10,811 (0.09)	1,397,442 (11.86)	34 (0)	10,318,386 (87.54)
RJC	10,289,838	10,247,413 (100)	64,104 (0.63)	2,469 (0.02)	5,252 (0.05)	6,82,430 (6.66)	40 (0)	9,493,118 (92.64)

Subsequently, small RNAs were classified into different categories according to their biogenesis and annotations (Table 2). Both RJM and RJC contained several heterogeneous small RNA species which included miRNAs, degraded rRNA fragments and mRNA fragments. In royal jelly, miRNAs were the major fraction of small RNA species. As shown in Table 3, the total reads of miRNAs in RJC (1,872,895 reads) were higher compared to RJM (1,735,052 reads).

**Table 2:** Different categories of small RNAs in RJM and RJC.

	RJM		RJC	
	Unique (%)	Total (%)	Unique (%)	Total (%)
Total sRNAs	1,176,366 (100.00)	10,318,386 (100.00)	1,310,750 (100.00)	9,493,118 (100.00)
miRNA	503 (0.04)	8,042 (0.08)	210 (0.02)	1,542 (0.02)
rRNAetc	335,464 (28.50)	3,969,740 (38.45)	324,232 (24.73)	3,476,557 (36.62)
unann	841,583 (71.46)	6,342,898 (61.47)	986,308 (75.25)	6,015,019 (63.36)

**Table 3:** Summary of common and specific sequences in RJM and RJC.

	Unique sRNAs (%)	Total sRNAs (%)
Total sRNAs	2,222,699 (100.00)	19,811,504 (100.00)
RJM specific	1,046,333 (47.07)	1,735,052 (8.76)
RJC specific	911,949 (41.03)	1,872,895 (9.45)
Shared RJM/RJC	264,417 (11.90)	16,203,557 (81.79)

**Figure 2:**
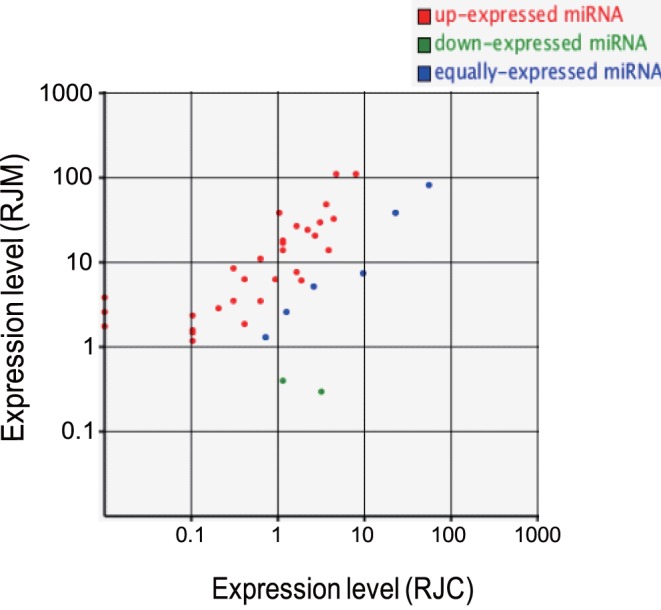
Differential expression analysis of miRNA in RJM and RJC. Expression levels were indicated on Y (RJM) or X (RJC). Expression levels were considered different if the threshold of false discovery rate (FDR was 0.001 and the absolute value of log2 Ratio was 1).

**Figure 3:**
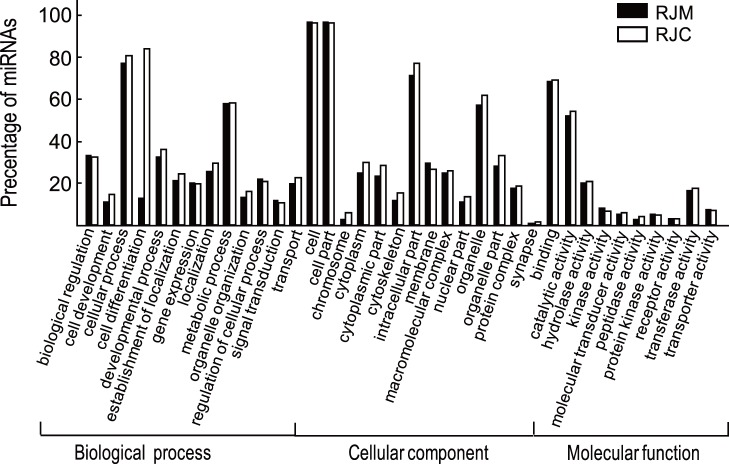
Gene Ontology classification of miRNAs in RJM and RJC. The results are summarized in three main categories: biological process, cellular component and molecular function. The Y-axis indicates the precentage of miRNAs in a category. The X-axis indicates category.

Solexa sequencing and RNA classification indicated that expression profiles of miRNAs in RJM and RJC are significantly different. By referencing to the mirBase release 13.0 [37], we identified 69 and 48 known miRNAs in RJM and RJC, respectively. There were 23 miRNAs specific to RJM, 2 miRNAs specific to RJC, and 46 shared in both RJ (Table 4). The average expression level of all miRNA in RJM was about 8-fold higher than that of RJC (Table S1). Among these, RJM contained 31 up-expressed, 6 equally expressed, and 2 down-expressed miRNAs compared to RJC (Fig. 2). According to sequence homology, we noticed a high-percentage of miRNA from categories of metabolic process, cell part and catalytic activity (Fig. 3). Cellular process, cell and binding terms were dominant.

**Table 4:** Summary of known miRNA in RJM and RJC.

**RJM specific (23)**
ame-mir-1, ame-mir-71, ame-mir-3719, ame-mir-7, ame-mir-1000, ame-mir-210, ame-mir-279, ame-mir-278, ame-mir-iab-4,-ame-mir-3049, ame-ame-mir-263b, ame-mir-3720, ame-mir-316, ame-mir-12, ame-mir-3783, ame-mir-279b, ame-mir-3747b, ame-mir-996, ame-mir-981, ame-mir-282, ame-mir-3732, ame-mir-137, ame-mir-932, ame-mir-305
**RJC specific (2)**
ame-mir-92a, ame-mir-379
**Shared RJM/RJC (46)**
ame-mir-100, ame-mir-3759, ame-mir-184, ame-mir-133, ame-mir-927, ame-mir-275, ame-mir-13b, ame-mir-277, ame-mir-29b, ame-mir-8, ame-mir-92b, ame-mir-283, ame-mir-927b, ame-mir-276, ame-mir-2, ame-mir-3718a, ame-mir-31a, ame-mir-3785, ame-mir-11, ame-mir-190, ame-mir-14, ame-mir-993, ame-mir-315, ame-mir-929, ame-mir-13a, ame-mir-2796, ame-mir-2944, ame-mir-263, ame-mir-375, ame-mir-124, ame-mir-87, ame-mir-34, ame-mir-750, ame-mir-989, ame-mir-3477, ame-mir-281, ame-mir-10, ame-mir-125, ame-mir-9a, ame-let-7, ame-mir-3786, ame-mir-252, ame-bantam, ame-mir-317, ame-mir-306

### 2. Transcriptome Modifications in *A. mellifera* by Two Different RJs

To test the hypothesis that miRNAs in RJM and RJC affect *A. mellifera* transcriptome, we determined the global transcriptomes of adult *A. mellifera* developed from larvae fed either with RJM (mRJM) or RJC (mRJC). The total number of reads for mRJM and mRJC were 48,971,186 and 49,358,642, respectively (Table 5). The distributions of perfectly matched reads to the honey bee genome in mRJM and mRJC were not significantly different (Contingency Table Analysis, X^2^ = 1.14, P>0.2), however, those uniquely matched to the genome in the two types of bees were significantly different (X^2^ = 150, P<0.0001). When the mapping was to the honey bee genes (which do not include intron and UTRs), both perfectly matched and uniquely matched reads were significantly differently distributed in mRJM and mRJC (X^2^>150, P<0.0001 in both cases).

**Table 5:** Summary of reads in mRJM and mRJC.

	mRJM		mRJC	
	reads number	%	reads number	%
Total Reads	48,971,186	100.00	49,358,642	100.00
Total BasePairs	4,407,406,740	100.00	4,442,277,780	100.00
**Mapped to Genome**				
Total Mapped Reads	38,461,269	78.54	38,759,459	78.53
Unique Match	37,970,456	77.54	38,127,557	77.25
Perfect Match	27,411,616	55.97	27,638,426	56.00
Total Unmapped Reads	10,509,917	21.46	10,599,183	21.47
**Mapped to Gene**				
Total Mapped Reads	31,019,818	63.34	31,629,813	64.08
Unique Match	28,160,531	57.50	28,732,144	58.21
Perfect Match	23,751,463	48.50	24,053,249	48.73
Total Unmapped Reads	17,951,368	36.66	17,728,829	35.92

**Figure 4:**
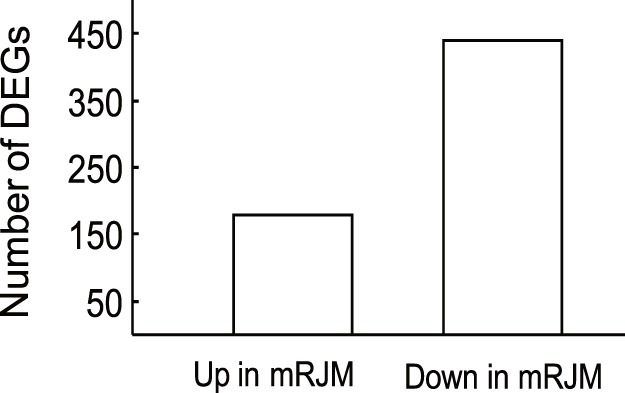
Differential expression analysis of genes in mRJM. Expression levels were considered different if the threshold of false discovery rate (FDR was 0.001 and the absolute value of log_2_ Ratio was 1).

**Figure 5:**
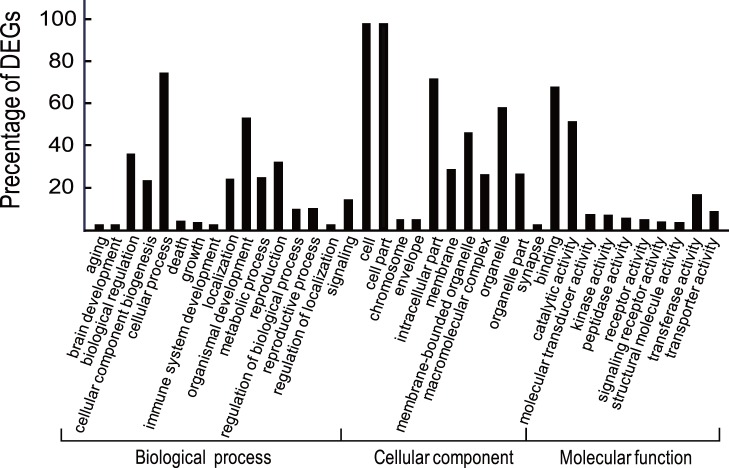
Gene Ontology classification of DEGs in mRJM. The results are summarized in three main categories: biological process, cellular component and molecular function. TheY-axis indicates the precentage of DEGs in a category. The X-axis indicates category.

By use of the Reads Per kb per Million reads (RPKM) method [38], we explored gene expression levels in mRJM and mRJC. This method was adopted to eliminate the influence of variation in gene length and the total reads number. The number of down-regulated genes was more than two times that of up-regulated genes, with 439 down-regulated and 179 up-regulated genes (Fig. 4, Table S2). We systematically examined every differentially expressed gene (DEG) in order to identify genes involved in important pathways. As shown in Table S3, these DEGs were mainly located in endocytosis, focal adhesion, metabolic pathways, regulation of actin cytoskeleton, and RNA transport. Some DEGs were related to pathways on caste differentiation, such as insulin signaling, mTOR, and MAPK. According to sequence homology, we obtained DEGs from categories in metabolic process, cell part and catalytic activity (Fig. 5). Cellular process, cell and binding terms were dominant.

### 3. Analysis of miRNA Targeted Genes

We identified the target genes of the following miRNAs: 23 RJM-specific, 2 RJC-specific and 33 differentially expressed miRNA (Table S4). Among the 618 differentially expressed genes between mRJM and mRJC, 144 (23.3%) genes were identified as target genes of miRNAs (Table S4).

## Discussion

### 1. miRNA Differences in RJ of *A. mellifera* and *A. cerana*

miRNA in honey bees have been shown to correlate with behavioral plasticity [27]–[29]. In our paper, we were more concerned with miRNAs in royal jelly that may play roles in affecting transcriptome. High-throughput sequencing of small RNAs indicated that there were many small RNAs in the two types of royal jelly (Fig. 1). Small RNAs are of 18–35 nt, which includes three major types: miRNA (20–22 nt), siRNA (24–26 nt), and piRNA (32–34 nt). RJC has a higher percentage of piRNA, especially those with 33 nt (Fig. 1).

We detected 23 unique miRNA in RJM and 2 in RJC. In addition, there were 33 miRNAs differentially expressed in the two types of RJ (Fig. 2). In the up-regulated miRNAs (Table S1), ame-bantam, ame-mir-184, ame-mir-14, ame-mir-252 were the most abundant in RJM. The four miRNAs (ame-let-7, ame-mir-34, ame-mir-100, ame-mir-375) commonly found in other animal bodies or products (such as milk [39] or humans [40] and mouse [41]) were also present in RJ. Only two miRNAs (ame-mir-10, ame-mir-2944) showed higher expression in RJC. Consistent with Guo [15], we also found ame-mir-263, ame-mir-277, and ame-mir-283 in the two types of RJ. However, RJM in our study contained ame-mir-263b, which was absent in their study. This might be due to the fact that our RJ was obtained from 3 day old larvae (largely 4th instar larvae [42]) while they obtained RJ from 4–6th instar larvae.

### 2. Transcriptome Modification in *A. mellifera* due to Two Different RJs

Though *A. mellifera* and *A. cerana* might diverge from a common ancestor, they show differences in morphology, physiology and disease resistance. After fed with heterospecific royal jelly, *A. mellifera* showed many DGEs. Kucharski et al. [43] proposed that important elements of glutamatergic synapses are G-protein coupled metabotropic glutamate receptors (GPC mGluRs), which contribute to synaptic plasticity and development. According to their sequence similarity, transduction mechanism and pharmacological profile, mGluRs are divided into three groups: group I (mGluR1 and mGluR5 receptors), group II (AmGluRA), and group III. The mGluR1 receptor links to phospholipase C, which causes phosphoinositide hydrolysis and release of calcium from intracellular stores. In mRJC, the expression level of mGluR1 (GB406151) was lower than that in mRJM, which might affect synaptic plasticity and development in the queen. Myosins [44] are one of three superfamilies of transporting motor proteins, which is involved in organelle formation, vesicle transportation, and cytoskeleton organization. In mRJC, Mhc1 (GB409843, a member of myosins) also decreased. The expression levels of InR-2 (GB725827), NLG-1 (GB724358), and trpgamma (GB410823) also decreased in mRJC. InR-2 is a member of insulin and insulin-like growth factor [45], which is linked to reproductive division of labor and foraging behavior [28]. NLG-1 and trpgamma are closely related to sensory input arising from environmental stimuli [46]–[48]. Four DEGs (GB552209, GB550937, GB410013, and GB409278) were involved in melanogenesis. They showed higher expression in mRJC and could explain prior studies showing darker coloration in mRJC [49], as was also the case in this study. *A. cerana* has been shown to have a higher sensitivity to odor than *A. mellifera* [50]. Seven genes (GB552209, GB406100, GB724316, GB551935, GB410013, and GB725569) involved in olfactory transduction were up-regulated in mRJC.

### 3. Analysis of miRNA Targeted Genes

miRNAs may specifically bind to partially complementary sites of targeted genes and inhibit the mRNA transcription. After feeding with RJM, 179 genes were up-regulated and 439 were down-regulated in *Apis mellifera.* Out of these 618 DEGs, 144 genes (67 up-regulated and 77 down-regulated) were putative targets of miRNA (Table S4), resulting a rather high 23%. The targeting of miRNAs on DEGs was not specific, with some miRNAs targeting more than 1,000 genes. Some DEGs only affected metabolic pathways (GB726969, GB726367, GB551389, GB100576257, GB724654, GB100577109, GB410748, GB551858, GB724644, GB726961 and GB551379), others were only involved with immune response (GB409078, GB100577433, GB412109, and GB725958), yet others had only limited participation in protein construction (GB100576328, GB410202, GB725868, and GB727133). Still, most DEGs participated in many pathways at the same time, such as GB552530, GB100577495, GB550937, and GB412869. Additionally, we found that some DEGs were involved in caste differentiation related pathways: insulin signaling (GB411959, GB100577495, GB412869, GB725827, GB552209, GB725200, GB724295, GB406096, GB410013, GB677665, and GB413596), Wnt signaling (GB552530, GB552021, GB100576682, GB550937, GB725891, GB724863, GB409278, GB726113, and GB725376), MAPK signaling (GB100577723, GB72624 7, GB725775, GB727172, GB725891, GB725987, GB725025, GB724732, GB410013, and GB100578991), and mTOR signaling pathway (GB412104 and GB100576439).

### General Conclusions

We show for the first time that miRNAs in royal jelly are different between *A. mellifera* and *A. cerana*. Further, transcriptomes are modified as a result of bees being fed royal jelly of different species. Because a high proportion of the differentially expressed genes were target genes from miRNA, we speculate that the transcriptome modifications are partly caused by miRNA differences of the two species. This study provides the first evidence that miRNA in heterospecific royal jelly can modify gene expression in honey bees. Our results suggested that royal jelly from *A. mellifera* and *A. cerana* have different epigenetic effect on gene expression, although these two species are evolutionarily closely related.

## Materials and Methods

Honey bee colonies (*Apis mellifera* and *Apis cerana*) were raised at the Honeybee Research Institute, Jiangxi Agricultural University, Nanchang, China (28.46°N, 115.49°E) by standard beekeeping techniques.

### 1. Differences in miRNA between Royal Jelly of Two Different Species of Honey Bees

#### Harvest of RJM and RJC

RJM and RJC were produced according to standard practices in China [51]. Briefly, the queen was confined inside a queen excluding cage. Queen cups with young larvae (one day old) were introduced into the colony and allowed to be fed by workers for 2 days. We first carefully removed 3 day old larvae by using either a grafting tool or a pair of forceps, then removed the royal jelly by using a spatula.

#### Measurement of miRNA between RJM and RJC

For miRNA analysis, freshly collected RJM and RJC (N = 4 samples per species, each with 100 mg RJ) were immediately extracted for total RNA. All four samples of each species RJ were pooled to create one sample for RJM and one for RJC. Total RNA was extracted using TRIzol reagent (Invitrogen, Carlsbad, CA, USA) according to manufacturer’s protocol. RNA quality was checked by using an Agilent 2100 Bioanalyzer. RNA fragments of 10–44 bases long were separated from total RNA by using Novex 15% TBE-Urea gel (Invitrogen), followed by 10% TBE-Urea gel. The resulting small RNAs were ligated to 5′ adaptors (Illumina, San Diego, CA, USA) and then combined with 3′adaptors (Illumina). These 5′ and 3′ products were amplified by PCR amplification and excised from 6% TBE-Urea gel (Invitrogen). According to Illumina Genome Analyzer (Beijing Genomics Institute, Shenzhen, China) instructions, the purified DNA segments were directly used for cluster generation and sequence. The sequencer produced image files were then converted to digital-quality data.

To further analyze the RNA secondary structure comprised with the matched Solexa reads, digital-quality sequences with perfect match or one mismatch were maintained. Genomic sequences of 100 nt were taken from these sequences, then the secondary structure was predicted and analyzed by RNAfold and MIREAP [39] under default settings. There are three criteria for candidate miRNA genes: (a) mature miRNAs are present in one arm of the hairpin precursors lacking large internal loops or bulges; (b) the secondary structures of the hairpins are steady, with the free energy of hybridization being lower than −20 kcal/mol; (c) hairpins are located in intergenic regions or introns [39]. Finally, these candidate miRNA reads were analyzed by miRBase database 13.0.

To compare miRNA expression levels between RJM and RJC, the reads of every miRNAs were subjected to the following analysis: a miRNA was considered “altered” only if it had both: (a) 10 copies by Solexa sequencing in both RJM and RJC, and (b) a two-fold difference in copy numbers between RJM and RJC. GO assignments usually has three ontologies: biological process, cellular component and molecular function, which is used to classify the functions of almost all miRNA in our paper.

### 2. Transcriptome Modification in *A. mellifera* Fed Two Different RJs

Honey bee (*A. mellifera*) larvae were reared inside 24-cell tissue culture plates (Costar, NY, USA) inside an incubator (35°C, 75±3% RH). Each cell was primed with 200 µl of freshly collected royal jelly, either from RJM or from RJC before a 1 day old larva was transferred into it. Larvae were transferred every 8 hrs to another plate with new food. For pupation, 6 day old larvae were transferred to 6-cell tissue culture plates lined with a piece of Kimwipe and kept in an incubator (35°C and 78±3% RH) [52]. After adult emergence, we obtained one sample per treatment (mRJM or mRJC), each consisted of 10 adult bees (5 from each of the two colonies) and used their heads for global transcriptome analysis. The two samples were kept at −80°C until use. A total of 1,400 larvae were reared for this experiment.

#### Measurement of transcriptomes in mRJM and mRJC

Total RNA was extracted with TRIzol regent (Invitrogen, USA) and treated with RNase-free DNase I (Takara Biotechnology, China). Poly(A) mRNA was separated by oligo-dT beads and then treated with the fragmentation buffer. By use of reverse transcriptase and random hexamer primers, the RNA fragments were transcribed into first-strand cDNA. Second strand cDNA synthesis was performed with DNA polymerase I and RnaseH. End-repair was done with T4 DNA polymerase, Klenow fragment, T4 Polynucleotide kinase. Ligation was accomplished with adapter or index adapter using T4 quick DNA ligase. Adaptor ligated fragments were selected according to size. Desired range of cDNA fragments were then excised from the gel. Finally, after validation of Agilent 2100 Bioanalyzer and ABI StepOnePlus Real-Time PCR System, the cDNA library was sequenced by Illumina HiSeq2000.

By use of SOAP [53], a specific transcript with uniquely mapped reads was counted and their sequences assembled. Mapped reads was evaluated according to RPKM (Reads Per kb per Million reads) value of each transcript [38]. The transcript fold change was then calculated by the formula of log_2_ (*m*RJM)_RPKM/(*m*RJC)_RPKM. The formula to calculate the probability of a specific gene being expressed equally between the two samples was defined as
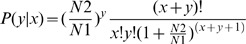
()Where N1 and N2 indicate the total number of clean reads in mRJC and mRJM, respectively, and x and y indicate the mapped clean read counts of the transcript in each sample respectively. Then, the FDR (False Discovery Rate) method was applied to determine the threshold of the p-value in multiple tests. In this study, ‘FDR<0.001’ and the absolute value of log_2_Ratio >1 were used as the threshold to judge the significance of differentiated gene expression. We used the Blastall program to annotate the pathways of DEGs against the KEGG database.

### 3. Analysis of miRNA Targeted Genes

To identify possible target sequences of RJ, we used the RNA hybrid software and ftp.ncbi.nih.gov/genomes/Apis_mellifera/RNA/rna.fa.gz, which provided us with a single predicted site of interaction with a minimum free energy.

## Supporting Information

Table S1 **The miRNAs expression analysis in RJM and RJC.**
(DOC)

Table S2 **Differential expressed genes (DEGs) analysis in mRJM, relative to mRJC.**
(DOC)

Table S3 **DEGs in biological process, cellular component, molecular function.**
(DOC)

Table S4 **Annotation of DEGs and their related miRNAs as affected by RJM and RJC.** Those that were up-methylated by RJM were indicated as bold, and those not bolded were down-regulated.
(DOC)
