# Supplementary material for: Epigenetic Modification of Gene Expression in Honey Bees by Heterospecific Gland Secretions
Source: PLoS One. 2012 Aug 21;7(8):e43727. doi: 10.1371/journal.pone.0043727 (PMC3424160; doi:10.1371/journal.pone.0043727)
Supplement: Table S1 — The miRNAs expression analysis in RJM and RJC. (DOC) [file pone.0043727.s001.doc]

Table S1. The miRNAs expression analysis in RJM and RJC.

| **Name** | **RJM-std** | **RJC-std** | **fold-change**  **(log2 RJM / RJC)** | **P-value** | **Sig-lable** | **Up/down**  **/equally** |
| --- | --- | --- | --- | --- | --- | --- |
| ame-bantam | 109.0287 | 4.8456 | 4.49188882 | 4.5987723917316e-251 | ** | Up |
| ame-let-7 | 6.1056 | 0.9481 | 2.68702194 | 2.24004601815843e-10 | ** | Up |
| ame-mir-100 | 7.4624 | 1.6854 | 2.14654866 | 7.21699849389486e-10 | ** | Up |
| ame-mir-125 | 1.5506 | 0.1053 | 3.88024923 | 0.0002792936681492 | ** | Up |
| ame-mir-133 | 3.3920 | 0.6320 | 2.42413971 | 9.67383388016649e-06 | ** | Up |
| ame-mir-14 | 26.5545 | 1.6854 | 3.97779343 | 1.68763996606283e-57 | ** | Up |
| ame-mir-184 | 109.3194 | 8.1111 | 3.75250806 | 8.04024893025885e-222 | ** | Up |
| ame-mir-2 | 2.8105 | 0.2107 | 3.73756460 | 7.73970201233378e-07 | ** | Up |
| ame-mir-210 | 2.5198 | 0.01 | 7.97716542 | 4.48514760654254e-08 | ** | Up |
| ame-mir-252 | 23.5502 | 2.2121 | 3.41225080 | 2.15492070047157e-45 | ** | Up |
| ame-mir-263 | 4.9904 | 1.0534 | 5.17250963 | 1.67237931413107e-95 | ** | Up |
| ame-mir-263b | 1.7797 | 0.01 | 8.56212792 | 9.30685506330434e-12 | ** | Up |
| ame-mir-275 | 6.0087 | 1.8961 | 1.66401784 | 3.26848070334625e-06 | ** | Up |
| ame-mir-276 | 47.0035 | 3.6869 | 3.67228800 | 1.08313825010385e-94 | ** | Up |
| ame-mir-277 | 6.2025 | 0.4214 | 3.87958761 | 3.74925595487082e-14 | ** | Up |
| ame-mir-2796 | 29.2681 | 3.1602 | 3.21124132 | 3.8726840352925e-53 | ** | Up |
| ame-mir-281 | 1.4537 | 0.1053 | 3.78715223 | 0.000508154232493374 | ** | Up |
| ame-mir-283 | 1.8414 | 0.4214 | 2.12754083 | 0.00299872978042514 | ** | Up |
| ame-mir-315 | 20.3520 | 2.7388 | 2.89355475 | 6.27634348493245e-34 | ** | Up |
| ame-mir-317 | 31.8848 | 4.5296 | 2.81541327 | 7.00911575500625e-51 | ** | Up |
| ame-mir-34 | 3.4889 | 0.3160 | 3.46477578 | 7.81026783787039e-08 | ** | Up |
| ame-mir-3720 | 1.7445 | 0.01 | 7.44666979 | 8.28365779056062e-06 | ** | Up |
| ame-mir-375 | 13.8588 | 3.8976 | 1.83014440 | 3.16081852097306e-14 | ** | Up |
| ame-mir-3759 | 8.3346 | 0.3160 | 4.72111650 | 6.0403129500594e-21 | ** | Up |
| ame-mir-3785 | 16.7662 | 1.1587 | 3.85497675 | 5.34398963666056e-36 | ** | Up |
| ame-mir-87 | 2.3259 | 0.1053 | 4.46521173 | 2.14604766396683e-06 | ** | Up |
| ame-mir-927 | 10.9513 | 0.6320 | 4.11503377 | 4.72975609598998e-25 | ** | Up |
| ame-mir-927b | 17.9292 | 1.1587 | 3.95173213 | 4.31115401150947e-39 | ** | Up |
| ame-mir-929 | 1.4537 | 0.1053 | 3.78715223 | 0.000508154232493374 | ** | Up |
| ame-mir-996 | 1.1630 | 0.1053 | 3.46527376 | 0.00300018896166747 | ** | Up |
| ame-mir-9a | 13.7618 | 1.1587 | 3.57009019 | 4.11004494053602e-28 | ** | Up |
| ame-mir-993 | 1.3877 | 1.1587 | -1.57949445 | 0.0528279075701067 |  | Equally |
| ame-mir-306 | 2.1365 | 2.6335 | 0.96380419 | 0.5894889897341069 |  | Equally |
| ame-mir-31a | 8.8686 | 9.6912 | -0.41499780 | 0.639203660812486 |  | Equally |
| ame-mir-3477 | 1.2599 | 0.7374 | 0.77278991 | 0.862270976557657 |  | Equally |
| ame-mir-750 | 1.5198 | 1.2641 | 0.99519863 | 0.851575027334306 |  | Equally |
| ame-mir-8 | 23.1843 | 23.2800 | 0.71388852 | 2. 210105208288867 |  | Equally |
| ame-mir-10 | 20.4389 | 56.4620 | -0.51061312 | 1.12951482804381e-10 | ** | Down |
| ame-mir-2944 | 0.2907 | 3.2655 | -3.48970093 | 1.2008548670392e-07 | ** | Down |
