# Supplementary material for: Epigenetic Modification of Gene Expression in Honey Bees by Heterospecific Gland Secretions
Source: PLoS One. 2012 Aug 21;7(8):e43727. doi: 10.1371/journal.pone.0043727 (PMC3424160; doi:10.1371/journal.pone.0043727)
Supplement: Table S2 — Differential expressed genes (DEGs) analysis in mRJM, relative to mRJC. (DOC) [file pone.0043727.s002.doc]

Table S2. Differential expressed genes (DEGs) analysis in mRJM, relative to mRJC.

| **Up-regulated genes in mRJM** |
| --- |
| 100578333, 100577443, 551272, 726094, 100576518, 412162, 726969, 100578034, 100577321, 100578297, 725163, 100576320, 411959, 725447, 100577882, 552543, 100576344, 100578861, 410063, 100578595, 100577186, 100576253, 100577630, 552530, 726367, 726046, 552561, 100576682, 550937, 550899, 413109, 726528, 100577777, 410202, 100577165, 100577015, 100577495, 725868, 100576470, 100576206, 100577107, 100576948, 100576723, 551917, 100578202, 100577432, 725233, 100578990, 726250, 724473, 100577549, 100576328, 727085, 100578410, 727546, 412869, 725506, 726905, 724783, 410220, 100577146, 408420, 550964, 100577193, 100577723, 100577448, 100577498, 724864, 100576781, 411019, 725404, 100576633, 100577009, 100578345, 100577848, 408830, 410623, 100577028, 100577831, 100577199, 100578987, 406151, 409843, 100576624, 100578066, 410736, 100576713, 725827, 406147, 408645, 100577072, 100578229, 725219, 100576277, 724410, 100577916, 408331, 413204, 100576090, 100577071, 412310, 724301, 551389, 100578457, 100577569, 100577030, 552311, 100577991, 725353, 408586, 724636, 100578094, 727611, 724358, 100576257, 724654, 413466, 100576449, 725668, 100578338, 100578134, 100577483, 551506, 100578317, 100576234, 551991, 410823, 726829, 725699, 726247, 100578091, 100578157, 725754, 551259, 100578267, 408807, 100577458, 100576129, 100577034, 409078, 100577433, 100578067, 552209, 725775, 725027, 100576496, 552021,100576288,100576128,100577109,100576703, 100578019,100576110, 408942, 726400, 411270, 724762, 725588, 411065, 100577283, 724980, 410748, 410994, 725200, 100578512, 551858, 100578484, 100578776, 411184, 409970, 724152, 100578231, 100577376, 410462, 100577900, 410259, 100578770, 551510, 412541 |
| **Down-regulated genes in mRJM** |
| 727172, 413908, 100576590, 100576409, 724158, 100578533, 412109, 100578387, 725675, 100577533, 727649, 100576082, 100578918, 100577773, 100576255, 100577836, 411353, 692346, 727133, 727142, 412192, 100579030, 100576570, 726711, 100577693, 100576515, 726900, 100578020, 100576918, 406100, 727419, 552743, 100576201, 100576195, 100577355, 100576560, 100577160, 727186 100578426, 552772, 100577506, 100578936, 100578152, 724284, 100577735, 724644, 409553, 726474, 100576779, 100576556, 725183, 100576902, 724316, 726515, 100578424, 726411, 724903, 100576152, 726261, 726232, 100577768, 727592, 412085, 726961, 100578966, 726315, 100578967, 100578002, 410747, 725891, 726894, 100578782, 725987, 551717, 551433, 100578674, 100576357, 552471, 410626, 725879, 725025, 100576641, 725958, 724295, 551539, 100577586, 100578574, 100577958, 727156, 551696, 724303, 100577644, 100578648, 100576979, 100577930, 100577950, 726016, 726627, 410753, 100578330, 413607, 724339, 725020, 100576677, 100576870, 727193, 100577840, 100578296, 551935, 725835, 724863, 724206, 411996, 100576531, 100576378, 406096, 100577459, 100576415, 100577480,552284,100576135,100577518,551908, 724732, 727507, 410013, 100576610, 100577279, 100578394, 100576348, 410107, 724249, 100578303, 552829, 725736, 100576734, 409278, 552425, 100578243, 677665, 726113, 551379, 100576436, 411321, 100577473, 100576758, 100577846, 726290, 725328, 409709, 724203, 100576655, 551601, 100578124, 725376, 726838, 100578479, 100578999, 551589,724312,411772,494506, 551374, 100577211, 552832, 725111, 724443, 412431, 411482, 100576126, 551193, 411675, 551094, 100577210, 725689, 406115, 100577143, 677673, 410370, 100578697, 100578542, 100577516, 551665, 725569, 100578693, 100578526, 411408, 551544, 100576322, 551759, 726530, 100576198, 100576158, 726766, 100576886, 725323, 551090, 410765, 100577980, 725685, 724943, 408681, 725866, 408934, 100576261, 724693,552472,100578271,724179, 725415,100577580,726571,724343,724472,411474,551171,725387,724151,552327,551728,100578175, 100578089, 412104, 100578389, 100577553,410906,412005,551055,727290,410743,724708,409644, 100577429,727568,725326,100576674,100576701,726859,100576708,100579040,726107,725213,406068, 726372,724269, 724211, 727074, 410509, 725031, 100577156, 678511, 409495, 552268, 724457, 100576389, 725620,100576163, 100576506, 411459, 726383, 726617, 551367, 100578818, 100578485, 726504, 550665, 100578329, 727539, 410884, 726020, 100577454, 551702, 100578559, 724534, 100577681, 552548, 411607, 726397, 408453, 412484, 100578995, 413596, 725458, 724995,412021 410201, 100576954, 727620, 412245, 100577247, 100578428, 100577566, 724730, 412277, 727031, 412112, 724126, 100578347, 100577648, 100576996, 413043, 552623,551273,100576143,551464,726890 100577946, 100577527, 411448, 727423, 411317, 552134, 408538, 724954, 100578713, 100577118, 100576937,100578750,552536, 725159, 727490, 410828, 725997, 550870, 100576274, 409638, 410675, 408805, 725272, 100578090, 24285,100579019,100577989,413705,552041,551080,552790,725114, 725429, 100578991, 724199,726874,100577931,408534,725140,411955,412562,724415, 100577192, 100576289,552383,551741551841, 409073, 724290, 725486, 677664, 408988,100577249,727401,725804, 725187, 726132, 725171,409798,408935,552028,412745,725484,551934,725397, 100578823,727180, 100578339, 725759, 724115, 409000, 411907, 100577649, 413930, 100578382, 726087, 726480,725146, 410755, 725215, 411670,409483,726373,724356,412448,100576133, 100578299, 100577809, 551237, 552373, 552399, 100576850, 725483, 726786, 727032, 552652, 724150, 552287, 413056, 100578129, 551608, 100577341, 100578563, 100578205,409422,551109,410070,727145,551375,406101,409469,410878,413040,552714,100576439,727398,410432, 552447, 100576155, 100577158, 727652, 727260, 551597, 726404, 724288, 411916, 726777, 725204, 100577239, 100577992, 100579061,726352,100577161 |
