# Supplementary material for: Epigenetic Modification of Gene Expression in Honey Bees by Heterospecific Gland Secretions
Source: PLoS One. 2012 Aug 21;7(8):e43727. doi: 10.1371/journal.pone.0043727 (PMC3424160; doi:10.1371/journal.pone.0043727)
Supplement: Table S3 — DEGs in biological process, cellular component, molecular function. (DOC) [file pone.0043727.s003.doc]

*Table S3.* *DEGs in biological process, cellular component, molecular function.*

| **Pathway** | **Differentially expressed genes** |
| --- | --- |
| **Biological process** | |
| Adherens junction | 412162, 100576682, 550937, 725404, 100577831, 725827, 726829, 726247, 100577034, 409278, 100576198, 413596, 412021, 100577527, 409000, 100576439 |
| Axon guidance | 100578034, 552530, 100578338, 726829, 725775, 725588, 726474, 724863, 410013, 100576348, 725376, 725866, 678511, 550870, 100576439 |
| B cell receptor signaling pathway | 725891, 725958, 410013, 100578389, 100576163, 100576439 |
| Cytokine receptor interaction | 100578267, 100578776, 552743, 100576415, 551544, 725685, 412005, 410509, 100576506, 100577239 |
| Calcium signaling pathway | 412869, 100576277, 552209, 724316, 725891, 551908, 410013, 724708, 551702, 725458, 100578991, 727145, 726404 |
| Cell adhesion molecules (CAMs) | 724358, 726411, 727592, 411321, 725213, 678511, 725620, 100577527, 724285 |
| Cell cycle | 100576682, 550937, 100577848, 724158, 100577586, 100577950, 100576870, 100576348, 724249, 409278, 677673, 552548, 100577931, 552652 |
| Chemokine signaling pathway | 552530, 100576723, 726829, 411270, 100578020, 552743, 100576902, 725891, 724863, 725376, 725866, 724472, 100576163, 100576439 |
| Citrate cycle (TCA cycle) | 551917, 726905, 100577283, 724732, 726838 |
| Dorso-ventral axis formation | 100576320, 100577199, 551589, 725111, 725620, 411907, 100576439, 726404 |
| ECM-receptor interaction | 725163, 100577777, 100576206, 724783, 551510, 727649, 100576918, 727507, 100576348, 411675, 100578175, 412104, 725213, 678511, 724457, 100578485, 725114, 677664, 100576155 |
| Endocytosis | 100578333, 100576344, 550937, 100578094, 100577433, 725775, 100576128, 100576703, 725588, 727172, 412109, 552743, 100578424, 727592, 100578967, 551717, 725025, 100578330, 725689, 100578542, 725685, 724693, 409644, 410509, 100576389, 100577454, 413596, 727031, 408988, 100576439, 100577239, 726404 |
| ErbB signaling pathway | 100578333, 726829, 100578020, 100576152, 725891, 724179, 100576163, 550870, 100576439, 100577239 |
| Focal adhesion | 551272, 725163, 552530, 100577777, 100576206, 100578410, 724783, 410220, 725404, 727611, 726829, 100577034, 551510, 727649, 100576515, 100576918, 725891, 724863, 727507, 100576348, 725376, 411675, 725866, 412104, 725213, 678511, 100576163, 100578485, 413596, 725458, 725114, 677664, 100576439, 100577239 |
| Gap junction | 100576277, 410994, 100576255, 100576902, 725891, 410755, 100576439, 100577239 |
| GnRH signaling pathway | 412869, 552209, 411270, 100576902, 725891, 410013, 100578394, 100578991, 100576439 |
| Insulin signaling pathway | 100578333, 411959, 100577495, 412869, 725827, 100577483, 552209, 725200, 100576515, 100576152, 724295, 406096, 410013, 677665, 724179, 412104, 100576163, 413596, 100576439 |
| Jak-STAT signaling pathway | 100578333, 550937, 100576206, 100577448, 100578267, 100577836, 100578020, 100576415, 409278, 551544, 725685, 410509, 100576163, 100576506 |
| Lysosome | 725447, 100577071, 409078, 100577283, 100578533, 411353, 552772, 726474, 726315, 724303, 100576979, 100577950, 409709, 100576126, 100578542, 100577516, 726571, 409495, 726617, 552536, 551080, 552383, 725215 |
| mTOR signaling pathway | 100576515, 100576152, 724179, 412104, 100576163, 100576439 |
| MAPK signaling pathway | 100577723, 100577483, 726247, 100578091, 725775, 724980, 100577376, 727172, 100578918, 100577693, 100577506, 100578966, 725891, 725987, 725025, 724732, 410013, 100578394, 100578999, 724179, 410906, 100578991, 727145, 100576439, 100577239, 100579061 |
| Metabolic pathways | 726969, 411959, 726367, 552561, 100576723, 551917, 100578410, 725506, 726905, 100577848, 100577071, 551389, 100576257, 725668, 100577483, 100577109, 408942, 100577283, 725200, 100578512, 551858, 100578770, 412541, 413908, 100576409, 100578387, 727649, 100578918, 100577836, 411353, 727186, 552772, 100577506, 100578936, 724644, 409553, 724316, 726961, 726315, 725891, 100578782, 551717, 100577586, 100576979, 413607, 724339, 100576870, 727193, 725835, 724863, 411996, 406096, 100576415, 724732, 100578394, 552829, 551379, 411321, 409709, 726838, 100578479, 724312, 677673, 410370, 100578542, 411408, 551544, 100576886, 725685, 724943, 408681, 724343, 552327, 100578089, 412005, 551055, 727290, 727568, 726859, 100579040, 724211, 100576389, 100576506, 726617, 727539, 410884, 551702, 408453, 100578995, 724995, 413043, 552623, 724126, 552134, 724954, 552536, 725159, 410828, 725997, 551841, 408988, 727401, 725804, 551934, 100578382, 726087, 727032, 406101, 409469, 410878, 725204, 726777, 100576133 |
| Melanogenesis | 550937, 412869, 552209, 100576902, 725891, 410013, 409278, 725140, 100576439 |
| Neuroactive ligand-receptor interaction | 724473, 100578410, 410623, 406151, 408645, 100576277, 100577030, 100576195, 724316, 100578648, 100577950, 100578296, 100576415, 100576135, 100576610, 100578693, 100578526, 100576158, 724708, 100576674, 726372, 551702, 100577118, 408534, 410755, 726352 |
| Neurotrophin signaling pathway | 100577495, 412869, 552209, 411270, 100577506, 100578966, 725025, 410013, 100578999, 724179, 100576163, 100576439, 551608 |
| Olfactory transduction | 412869, 100577448, 552209, 100576255, 724316, 100577768, 100577840, 410013, 725569, 724708, 100576954, 725429, 727401, 725759, 551237, 409422, 406100, 551935 |
| Oocyte meiosis | 100577015, 412869, 100577498, 552209, 724158, 100576902, 100577586, 100577950, 410013, 100576348, 724179, 413596, 552041, 411907, 100576439, 100576155 |
| Oxidative phosphorylation | 551917, 100577506, 100578782, 413607, 100576886, 408681, 412005, 726617, 726777 |
| p53 signaling pathway | 100576206, 100578410, 100577458, 726020, 100578347, 100576937, 100577931, 406101 |
| Pancreatic secretion | 724410, 409553, 100576902, 725891, 100578394, 100576158, 100576674, 726372, 410884, 726890, 408534, 725759, 727032, 726352 |
| Peroxisome | 100577483, 412541, 100576255, 100578020, 100576902, 551717, 725025, 677673, 551171, 100578329, 552790, 725187, 410878, 413056 |
| Phagosome | 100576206, 725404, 100577034, 100576128, 410994, 100578533, 412109, 727592, 725689, 412104, 409644, 678511, 409495, 726617, 100577454, 100576439 |
| Phosphatidylinositol signaling system | 100578410, 412869, 552209, 100576409, 727649, 725891, 100577586, 406096, 551908, 410013, 411408, 100576389, 100576163, 551702 |
| Phototransduction | 412869, 725404, 410823, 100577034, 552209, 725891, 100576870, 551908, 410013 |
| PPAR signaling pathway | 412541, 100576515, 551717, 727193, 410884, 100578428, 409798, 727032 |
| Progesterone-mediated oocyte maturation | 100577015, 724158, 100576902, 100577586, 100576348, 724249, 724179, 724708, 100576163, 413596, 411907, 100576439 |
| Regulation of actin cytoskeleton | 100576344, 552530, 726250, 410220, 725404, Mhc1, 727611, 726247, 100577034, 100576128, 100576082, 100576779, 725183, 725987, 724863, 100576348, 100578124, 725376, 725866, 724472, 410906, 725213, 678511, 100576163, 725458, 410201, 100577648, 100576143, 551464, 724415, 410070, 100576439, 100577239 |
| RIG-I-like receptor signaling pathway | 100578034, 100577900, 727592, 725025, 551601, 100578693 |
| Salivary secretion | 725163, 100577777, 412869, 552209, 100576255, 100576902, 725891, 410013, 725114 |
| Taste transduction | 100576918, 725569 |
| T cell receptor signaling pathway | 100578333, 726829, 411270, 725958, 410013, 725620, 100576163, 550870, 100576439 |
| TGF-beta signaling pathway | 552530, 100576682, 550937, 100576206, 100578776, 100576152, 724863, 409278, 725376, 725866, 724179, 725415, 100576389, 408988, 100576439, 100577161 |
| Tight junction | 726250, 100578410, 725404, 100577831, 409843, 100577034, 100576128, 411270, 100576779, 725891, 551601, 100578124, 410201, 100577648, 100576143, 551464, 724285, 100578991, 677664, 100578299, 410070 |
| Toll-like receptor signaling pathway | 100578966, 725025, 100577518, 412104, 100576163, 100578347, 100576439, 551608 |
| Vascular smooth muscle contraction | 552530, 726250, 412869, 410220, 550964, 409843, 552209, 100576128, 411270, 100576255, 100576779, 100576902, 725891, 725987, 724863, 410013, 100578394, 100578124, 725376, 725866, 410906, 725458, 410201, 100577648, 100576143, 551464, 100578991, 410070, 100576439 |
| VEGF signaling pathway | 725891, 410013, 100578394, 100576163, 100576439 |
| Wnt signaling pathway | 410063, 552530, 100576682, 550937, 100578338, 552021, 100576082, 725891, 724863, 410013, 409278, 726113, 725376, 725866 |
| **Cellular component** | |
| Amino and nucleotide sugar metabolism | 726046, 726530, 100577156, 412245, 100577247, 413705, 725759, 100578382, 726480 |
| Aldosterone-regulated sodium reabsorption | 100577495, 725827, 100576515, 725891, 100576163, 413596, 100576439 |
| Arginine and proline metabolism | 100576870, 551094, 408988 |
| Biosynthesis of unsaturated fatty acids | 724654, 725031, 724126, 100577192, 725146 |
| Carbohydrate digestion and absorption | 725891, 100576163, 100578991 |
| Cysteine and methionine metabolism | 552561, 100578770, 724954, 725204 |
| Fatty acid biosynthesis and metabolism | 411959, 725200, 412541, 551717 |
| Fructose and mannose metabolism | 408681 |
| Galactose metabolism | 100576723, 551934 |
| Glutathione metabolism | 406101 |
| Glycolysis / Gluconeogenesis | 725506, 726905, 100577283, 408681 |
| GPI -anchor biosynthesis | 724339, 724943, 408681 |
| Glycine, serine and threonine metabolism | 726367, 725506, 726905, 100577283, 410748, 552425, 552832, 410743, 410432, 411916 |
| Glycerolipid metabolism | 726367, 100576409, 727649, 411353, 409553, 727193, 725685, 551055, 727539, 410884, 551702, 724995, 727032 |
| Glycerophospholipid metabolism | 410736, 725668, 100576409, 727649, 411353, 552772, 100578394, 552829, 724443, 100578542, 411408, 725685, 408934, 100576506, 727539, 551702, 724995 |
| Histidine metabolism | 725506, 100577283 |
| Inositol phosphate metabolism | 100578410, 100577586, 406096, 411408, 100576389, 100576163 |
| Lysine degradation | 100578091, 408942, 100577160, 724249, 678511, 412021, 100578991 |
| Metabolism of cytochrome P450 | 725506, 413908, 724863, 100576415, 408453, 413043, 552623, 725159, 725997 |
| N-Glycan biosynthesis | 100577109, 727186, 724644, 724316, 551379, 552327, 412005, 727568, 551841, 726087, 552373 |
| Pantothenate and CoA biosynthesis | 724312, 100578995 |
| Pentose and glucuronate interconversions | 724863, 100576415, 413043, 552623, 725997 |
| Protein digestion and absorption | 100576344, 724410, 551510, 724316, 411675, 551759, 100576158, 100576674, 726372, 100578485, 551273, 408534, 552028, 726786, 726352 |
| Purine metabolism | 100576518, 100578410, 550964, 100577009, 408942, 100577836, 552772, 100576902, 725835, 411996, 100578479, 677673, 410370, 725685, 724343, 100578089, 725804, 406101 |
| Retinol metabolism | 100578861, 413908, 724863, 100576415, 408453, 413043, 552623, 725159, 725997, 100578382 |
| Riboflavin metabolism | 100577516, 726571, 552134, 727401, 725215 |
| Sulfur metabolism | 552561, 100578410, 677673 |
| Tryptophan metabolism | 551858, 551094, 724126, 410828, 100576133 |
| Ubiquitin mediated proteolysis | 100578333, 412162, 725506, 100578094, 724654, 100577483, 100578157, 100578484, 100578918, 551717, 725025, 100577586, 677665, 551193, 410370, 100576198, 411459, 551367, 726020, 724954, 100576937, 727490, 552714, 551597 |
| Valine, leucine and isoleucine degradation | 726905, 100577283, 725891 |
| Vitamin digestion and absorption | 411019, 725668, 409553, 410626, 552829, 100576655, 724312, 412431, 551665, 410884, 100578995, 100577158 |
| **Molecular function** | |
| Aminoacyl-tRNA biosynthesis | 100577483, 725987, 100577279, 410370, 100578175, 724199, 725759, 100576155 |
| Basal transcription factors | 726474, 100577950, 410753, 550665, 408538, 100577341 |
| DNA replication | 100577836, 726711, 552772, 725835, 411996, 100578479 |
| Homologous recombination | 726250, 100577836, 100576560, 100578674, 100576677, 411996, 100576348, 100578479, 725685, 551464 |
| mRNA surveillance pathway | 726094, 552543, 100576253, 100577448, 100577498, 100577028, 726247, 408942, 100577836, 100576389, 412277, 100578991, 725397 |
| Mismatch repair | 100576344, 724152, 100577836, 411996, 100578479, 100577681 |
| Nucleotide excision repair | 100577836, 552772, 100577586, 725835, 411996, 725376, 100578479, 726020, 726397, 100578299, 100577341 |
| Ribosome and Ribosome biogenesis | 100577882, 552471, 726094, 552530, 726046, 100577495, 100576713, 100577458, 100578484, 727419, 100576378, 100577459, 725736, 724203, 725376, 406115, 100578693, 100576886, 724472, 100576701, 724534, 100578347, 100576996, 100577931, 100576850 |
| RNA degradation | 726046, 724284, 100576556, 552284, 410884, 410675, 725486, 413930, 727398 |
| RNA polymerase | 100578410, 410370, 724343, 100578089, 725804 |
| RNA transport | 552543, 410202, 100577146, 100577498, 100577028, 100576288, 100578770, 727649, 100577836, 727133, 727419, 100576641, 100577644, 100577950, 100578330, 100576378, 551908, 100577279, 100576348, 677665, 725376, 100576126, 411474, 408538, 724285, 100578991, 726874, 100577931, 677664, 727398, 724288, 727260 |
| Spliceosome | 725868, 100576328, 100577146, 100576633, 100578345, 100578770, 727172, 727649, 727133, 100578152, 724903, 100577279, 100576436, 410201, 100578750, 100578991, 725804 |
