# Supplementary material for: Epigenetic Modification of Gene Expression in Honey Bees by Heterospecific Gland Secretions
Source: PLoS One. 2012 Aug 21;7(8):e43727. doi: 10.1371/journal.pone.0043727 (PMC3424160; doi:10.1371/journal.pone.0043727)
Supplement: Table S4 — Annotation of DEGs and their related miRNAs as affected by RJM and RJC. Those that were up-methylated by RJM were indicated as bold, and those not bolded were down-regulated. (DOC) [file pone.0043727.s004.doc]

Table S4. Annotation of DEGs and their related miRNAs as affected by RJM and RJC.

| **Gene ID** | **Full name** | **The related miRNA** | **Gene Annotation / Pathway** |
| --- | --- | --- | --- |
| **551272** | talin-2-like | mir-277; mir-87; iab-4; mir-3049 | Focal adhesion |
| **412162** | armadillo repeat-containing protein 4-like | mir-3719; mir-3720; mir-305 | Ubiquitin mediated proteolysis; Adherens junction |
| **726969** | farnesyl pyrophosphate synthase- like | miR-927; mir-305; mir-92a | Metabolic pathways |
| **411959** | fatty acid synthase-like | mir-927b; mir-996; iab-4; mir-71; mir-996; mir-1; mir-3720; mir-3732 | Metabolic pathways; Insulin signaling pathway |
| **410202** | radial spoke head protein 3 homolog | mir-184 | RNA transport |
| **552561** | protein FAM188B-like | mir-263 | Metabolic pathways |
| **552530** | NGFI-A-binding protein homolog | let-7; mir-3049; mir-316; iab-4 | Regulation of actin cytoskeleton; Wnt signaling pathway |
| **726367** | glycerate kinase-like | mir-252; mir-263b; mir-2944; mir-927b; mir-278; mir-305; iab-4; mir-71; mir-932 | Metabolic pathways |
| **726046** | calmodulin-lysine N-methyltransferase-like | mir-3720; mir-278; mir-3720 | Metabolic pathways; Ribosome biogenesis |
| **100576682** | uncharacterized | mir-315 | Cell cycle; Wnt signaling pathway |
| **550937** | apolipoprotein D-like | mir-927; mir-87; mir-3732; mir-7; mir-278; mir-92a | Melanogenesis; Wnt signaling pathway |
| **410202** | radial spoke head protein 3 homolog | mir-137; mir-1000; mir-3719; mir-3783; mir-379 | RNA transport |
| **100577015** | uncharacterized | mir-927; mir-927b; mir-137; mir-316 | Progesterone oocyte maturation; Oocyte meiosis |
| **100577495** | uncharacterized | mir-315; mir-283; mir-125; bantam; mir-3049; mir-3747b; mir-1000; mir-71; iab-4; mir-7; mir-278 | Adipocytokine signaling pathway; Insulin signaling pathway; Ribosome biogenesis |
| **725868** | uncharacterized | mir-3785; miR-929; mir-932 | Spliceosome |
| **100576206** | uncharacterized | mir-996; mir-2; mir2796; mir-281; mir-87; mir-271; mir-996 | p53 signaling pathway; Phagosome |
| **551917** | Succinate dehydrogenase iron-sulfur subunit, mitochondrial-like | mir-2944; mir-927; mir-927b; mir-137 | Metabolic pathways; TCA cycle |
| **726250** | uncharacterized | mir-2944; mir-927; mir-927b | Tight junction; Regulation of actin cytoskeleton |
| **724473** | rhodopsin like | mir-375; mir-263b; mir-263; mir-316 | Neuroactive ligand-receptor interaction |
| **100576328** | uncharacterized | mir-283; mir-87; mir-927; mir-927b; mir-981; mir-7; mir-12 | Spliceosome |
| **412869** | Sarcoplasmic calcium-binding protein （CBP） | mir-2944; mir-283; mir-2; mir-279; mir-996; mir-1000; mir-981; mir-279b | Insulin signaling pathway; Melanogenesis; Salivary secretion |
| **725506** | uncharacterized | mir-263b; mir-275 | Metabolism of cytochrome P450; Metabolic pathway |
| **726905** | uncharacterized protein C20orf26- like | mir-277; mir-281; mie-283; mir-3759; mir-3783; mir-1; mir-1000; mir-932; mir-137; iab-4; mir-305; mir-122;mir-92a | Metabolic pathways; TCA cycle |
| **724783** | uncharacterized | mir-12 | Focal adhesion; ECM-receptor interaction |
| **410220** | uncharacterized | mir-87; mir-929; mir-278; mir-305; mir-3719; mir-3783 | Regulation of actin cytoskeleton; Focal adhesion |
| **550964** | guanylate cyclase 32E-like | mir-263; mir-929; mir-3732; mir-3049; mir-1000; mir-137 | Vascular smooth muscle contraction; Purine metabolism |
| **100577723** | uncharacterized | mir-929; mir-3785; mir-375; mir-263b; mir-282; mir-71; mir-3049; mir-379 | MAPK signaling pathway |
| **411019** | neuropilin and tolloid-like protein 2-like | mir-125; mir-3747b; mir-137 | Vitamin digestion and absorption |
| **725404** | actin, indirect flight muscle-like | mir-3720; mir-3720; mir-1;mir-379 | Regulation of actin cytoskeleton; Phagosome |
| **100576633** | uncharacterized | mir-392 | Spliceosome; Protein processing |
| **100577009** | uncharacterized | mir-3783 | Purine metabolism |
| **410623** | uncharacterized | mir-929; mir-87; mir-315; mir-3049; mir-3747b; mir-981 | Neuroactive ligand-receptor interaction |
| **406151** | metabotropic glutamate receptor 1(mGlutR1) | mir-227; mir-276; mir-263b; mir-2; mir-184; mir-14; let-7; mir-3049; mir-71;mir-379 | Neuroactive ligand-receptor interaction |
| **409843** | myosin heavy chain 1（Mhc1） | let-7; mir-100; mir-2; mir-2944; mir-315 | Regulation of actin cytoskeleton; Tight junction |
| **410736** | uncharacterized | mir-184; let-7; mir-3732;mir-3783;mir-3049;mir-3747b;mir-1; mir-981;mir-71;iab-4;mir-7; mir-379 | Glycerophospholipid metabolism |
| **100576713** | uncharacterized | mir-283; mir-927; mir-927b | Ribosome biogenesis |
| **725827** | insulin-like receptor-like（InR-2） | mir-2796; mir-263b; mir-14; mir-1;mir-3783 | Insulin signaling pathway; Adherens junction |
| **408645** | uncharacterized | iab-4 | Neuroactive ligand-receptor interaction |
| **100576277** | uncharacterized | mir-281; mir-125; mir-137 | Gap junction; Calcium signaling pathway |
| **724410** | uncharacterized | mir-34; mir-3720; mir-7; mir-3720;mir-379 | Protein digestion and absorption; Pancreatic secretion |
| **100577071** | uncharacterized | mir-263; mir-184; mir-100; let-7; mir-3732; mir-3719; mir-3049; mir-3747b | Lysosome; Metabolic pathways |
| **408586** | hormone receptor-like in 46（Hr46） | mir-3732; mir-278 |  |
| **551389** | apolipoprotein D-like | mir-133; mir-87 | Metabolic pathways |
| **100578094** | uncharacterized | mir-2944; mir-283; mir-263; mir-2; mir-14; mir-305; iab-4; mir-279; mir-932; mir-316 | Endocytosis; Ubiquitin mediated proteolysis |
| **727611** | uncharacterized | mir-315; mir-929; mir-278 | Regulation of actin cytoskeleton; Focal adhesion |
| **724358** | neuroligin 1（NLG-1） | mir-375; mir-315; mir-263; mir-125; mir-100; let-7; mir-1; mir-12; mir-137; mir-932; mir-3719 | Cell adhesion molecules (CAMs) |
| **100576257** | uncharacterized | mir-14; mir-315; mir-3719; mir-92a | Metabolic pathways |
| **725668** | phospholipase B1, membrane-associated-like | mir-283 | Metabolic pathways; Vitamin digestion and absorption |
| **724654** | cytochrome b5 type B-like | mir-305 | Metabolic pathways |
| **410823** | transient receptor potential-gamma protein（trpgamma） | mir-276; mir-210; bantam; mir-1; mir-210; mir-278; mir-71; mir-3732 | Phototransduction - fly |
| **726247** | uncharacterized | mir-133; mir-210; mir-281; mir-9a; mir-305; mir-210; mir-379 | mRNA surveillance pathway; MAPK signaling pathway |
| **409078** | uncharacterized | mir-929; mir-87; mir-3785; mir-277; mir-278; mir-7; mir-3732 | Lysosome |
| **100577433** | uncharacterized | mir-263; bantam; iab-4; mir-12 | Endocytosis |
| **552209** | calmodulin-like | bantam; mir-92a | Insulin signaling pathway; GnRH signaling pathway; Melanogenesis; Olfactory transduction |
| **725775** | uncharacterized | mir-133; mir-210; mir-34; | Endocytosis; MAPK signaling pathway; Axon guidance |
| **552021** | intraflagellar transport 80 homolog（IFT80） | mir-2944; mir-2; let-7; bantam | Wnt signaling pathway |
| **100577109** | uncharacterized | mir-133; mir-276; mir-315; mir-3720; mir-87 | Metabolic pathways |
| **408942** | uncharacterized | mir-3785; mir-2796; mir-263; mir-981; mir-7; mir-397 | Metabolic pathways; mRNA surveillance pathway |
| **411270** | protein kinase C delta（Pkcdelta） | mir-275; mir-2944; mir-34; mir-305; mir-7; iab-4; mir-279; mir-981; mir-316 | T cell receptor signaling pathway; Chemokine signaling pathway; Adipocytokine signaling pathway |
| **725588** | uncharacterized | mir-2944; mir-263b; mir-2; mir-133; mir-3049; mir-981 | Endocytosis; Axon guidance |
| **410748** | GMC oxidoreductase（GMCOX1） | mir-315; mir-996 | Metabolic pathways |
| **410994** | beta-Tubulin at 60D ortholog | mir-263b; mir-184; mir-125; let-7; mir-3747b; mir-981; mir-137; mir-379 | Phagosome; Gap junction |
| **725200** | IQ and AAA domain-containing protein 1-like | mir-14; mir-2; mir-263b; mir-281; mir-2944; | Metabolic pathways; Insulin signaling pathway |
| **551858** | kynurenine 3-monooxygenase（cn） | mir-277; mir-276; mir-263; mir-184; bantam; mir-305; mir-71; mir-3732; mir-92a | Metabolic pathways |
| **724152** | uncharacterized | bantam; mir-316; mir-1000; mir-137; iab-4 | Mismatch repair |
| **551510** | uncharacterized | mir-263; mir-929; mir-3719; mir-3049; mir-1000; mir-71; mir-379 | Protein digestion and absorption |
| **412541** | long-chain-fatty-acid--CoA ligase 1-like | mir-929; mir-283; mir-2796; mir-210; mir-184; mir-125; mir-305; mir-210 | Metabolic pathways; Adipocytokine signaling pathway |
| 727172 | uncharacterized | mir-87; mir-9a; mir-1000; mir-981 | MAPK signaling pathway |
| 413908 | cytochrome P450 6AS12（CYP6AS12） | mir-2944; mir-2796; mir-2; mir-184; mir-133; mir-1; mir-12; mir-137; mir-3783 | Metabolism of cytochrome P450; Metabolic pathways |
| 724158 | uncharacterized | mir-34 | Progesterone-mediated oocyte maturation; Cell cycle |
| 412109 | coiled-coil domain-containing protein 147-like | mir-315; mir-283; mir-276; mir-14; bantam | Endocytosis |
| 727649 | uncharacterized | mir-276; mir-283; mir-315; mir-927; mir-927b; mir-9a; mir-316; mir-282 | Metabolic pathways; Phosphatidylinositol signaling system |
| 411353 | lipase 3-like | mir-87; mir-3720; mir-34; mir-315; mir-277; mir-263 | Metabolic pathways; Lysosome |
| 727133 | sine oculis-binding protein homolog | let-7; mir-263; mir-315; mir-927; mir-927b; mir-12; mir-137; mir-316 | RNA transport; Spliceosome |
| 100576570 | uncharacterized | mir-87; mir-375; mir-263b; mir-210; mir-184; mir-210; mir-278; mir-137; mir-1000 | Metabolic pathways |
| 726711 | ribonuclease H2 subunit C-like | mir-1; mir-316; mir-981; mir-137 | DNA replication |
| 406100 | odorant binding protein 5（Obp5） | mir-263b; mir-3759; mir-927; mir-927b; mir-12 | Olfactory transduction |
| 727419 | ribonuclease P protein subunit p29-like | mir-283; mir-252; mir-1; mir-316; mir-305 | Ribosome biogenesis; RNA transport |
| 552743 | trafficking protein particle complex subunit 2-like protein-like | mir-276; mir-929; mir-1; mir-137; mir-92a | Chemokine signaling pathway; Endocytosis |
| 100578152 | uncharacterized | mir-996; mir-9a; mir-184; mir-263; mir-263b; mir-275; mir-283; mir-315; mir-317 mir-1; mir-1000; mir-996; mir-279; mir-305 | Spliceosome |
| 724284 | uncharacterized | mir-276; mir-1; mir-12; mir-282; mir-3719; mir-3783 | RNA degradation |
| 724644 | uncharacterized | mir-87 | Metabolic pathways |
| 409553 | pancreatic triacylglycerol lipase-like | mir-375; mir-317; mir-2796; mir-275; mir-263;mir-252; mir-14 | Metabolic pathways; Vitamin digestion and absorption |
| 726474 | PSEA-binding protein 45kD（Pbp45） | let-7; mir-210; mir-276; mir-277; mir-283; mir-87; mir-927b; mir-929; mir-210 | Lysosome; Axon guidance; Basal transcription factors |
| 725183 | probable palmitoyltransferase ZDHHC24-like | mir-927b; mir-927; mir-2944; mir-277; mir-275; mir-2; mir-137 | Regulation of actin cytoskeleton |
| 724316 | transmembrane protein 145-like | mir-252; mir-927; mir-927b; mir-3747b; mir-932; mir-7 | Metabolic pathways; Olfactory transduction |
| 726411 | hemicentin-1-like | mir-283; mir-276; mir-14; mir-7; iab-4; mir-1000; mir-92a | Cell adhesion molecules (CAMs) |
| 727592 | uncharacterized | mir-14; mir-263; mir-283; mir-315; mir-279b; mir-3783; mir-3747b; mir-137; mir-305 | Endocytosis; Antigen processing and presentation; CAMs |
| 412085 | post-GPI attachment to proteins factor 3 | mir-315; mir-2944; mir-263; mir-2; mir-71; mir-981; mir-12 | ABC transporters |
| 726961 | uncharacterized | mir-315; mir-927b | Metabolic pathways |
| 726315 | sphingomyelin phosphodiesterase 1-like | mir-2944; mir-283; mir-281; mir-2; mir-316 | Metabolic pathways; Lysosome |
| 100578967 | uncharacterized | mir-315; mir-9a | Endocytosis |
| 725891 | synaptotagmin 12（Syt12） | mir-996;mir-929;mir-927b;mir-927;mir-3720;mir-34; mir-14; mir-125; mir-12; mir-305; mir-137; mir-71; mir-996; mir-3720; mir-3783 | MAPK signaling pathway; ErbB signaling pathway; GnRH signaling pathway; Melanogenesis; Wnt signaling pathway |
| 725987 | uncharacterized | mir-14; mir-252; mir-283; mir-315; mir-9a; mir-3747b; mir-1; mir-316; mir-137; mir-305 | MAPK signaling pathway; Long-term depression; Regulation of actin cytoskeleton |
| 551717 | uncharacterized | mir-375; mir-315; mir-281; mir-1000 | Adipocytokine signaling pathway; Metabolic pathways |
| 552471 | mitochondrial ribosomal protein L52 （mRpL52） | mir-275 | Ribosome |
| 410626 | sodium-coupled monocarboxylate transporter 2-like | mir-996; mir-87; mir-184; mir-125; iab-4; mir-279; mir-71; mir-996; mir-1000; mir-279b | Vitamin digestion and absorption |
| 725025 | peroxisome biogenesis factor 10-like | mir-1000 | Endocytosis; Peroxisome; MAPK signaling pathway |
| 725958 | lymphoid tissue lymphoma translocation protein 1-like | mir-263b; mir-281; mir-315; mir-87; mir-927b; mir-996; mir-9a; mir-3783; mir-279b; iab-4; mir-279; mir-996; mir-92a | B and T cell receptor signaling pathway |
| 724295 | protein phosphatase 1 regulatory subunit 3C-B-like | mir-317; mir-276; mir-137 | Insulin signaling pathway |
| 551539 | uncharacterized | mir-277; mir-283; mir-1000 | NOD-like receptor signaling pathway |
| 727156 | vesicle transport protein USE1 | mir-927b; mir-316; mir-305; mir-137 | SNARE interactions in vesicular transport |
| 551696 | thioredoxin domain-containing protein 9-like | mir-9a; mir-375; mir-316; mir-12; mir-92a | Protein processing in endoplasmic reticulum |
| 724303 | AP-1sigma protein（AP-1sigma） | mir-316; mir-92a | Lysosome |
| 410753 | TATA-box-binding protein-like | mir-996; mir-927b; mir-927; mir-276; mir-282; mir-137; mir-279; mir-996; mir-92a | Basal transcription factors |
| 413607 | golgin-84 | mir-3783; mir-305; mir-7; mir-71; mir-92a | Oxidative phosphorylation; Metabolic pathways |
| 724339 | phosphatidylinositol N-acetylglucosaminyl transferase subunit Q-like | mir-1000; mir-316; mir-3049; mir-3719; mir-12; iab-4 | Metabolic pathways; Glycosylphosphatidylinositol-anchor biosynthesis |
| 100576870 | uncharacterized | mir-210; mir-275; mir-3759; mir-137; mir-316; mir-210; mir-7 | Metabolic pathways; Cell cycle |
| 100578296 | probable G-protein coupled receptor AH9.1-like | mir-263; mir-277; mir-87 | Neuroactive ligand-receptor interaction |
| 727193 | pancreatic triacylglycerol lipase-like | mir-71; mir-981; mir-316; mir-1; mir-3719; mir-3732; mir-305 | Metabolic pathways; PPAR signaling pathway |
| 551935 | odorant binding protein 21（Obp21） | mir-927; mir-927b; mir-12 | Olfactory transduction |
| 725835 | DNA polymerase epsilon subunit 4-like | mir-277; mir-305; mir-1000; mir-92a | Nucleotide excision repair; DNA replication |
| 724863 | uncharacterized | mir-210; mir-210; mir-137 | Wnt signaling pathway; Metabolism of cytochrome P450; Regulation of actin cytoskeleton; Axon guidance |
| 724206 | WD repeat-containing protein 67-like | mir-263; mir-277; mir-1000; mir-316; mir-3747b; mir-3049; iab-4; mir-3732 | Protein processing in endoplasmic reticulum |
| 411996 | uncharacterized | mir-283; mir-2944; mir-315; mir-87; mir-927b; mir-1000; mir-3747b | Homologous recombination; Mismatch repair; DNA replication |
| 100576378 | ribonuclease P protein subunit p30-like | mir-263b; mir-133; mir-12; mir-1000 | Ribosome biogenesis ; RNA transport |
| 406096 | type I inositol 1,4,5-trisphosphate 5-phosphatase | bantam; mie-14; mir-263b; mir-283; mir-9a; mir-3783;mir-12;mir-278;mir-7;mir-71 | Metabolic pathways; Insulin signaling pathway |
| 552284 | scavenger mRNA-decapping enzyme DcpS-like | mir-87; mir-315; mir-277; mir-71; mir-1000; mir-316 | RNA degradation |
| 551908 | uncharacterized | mir-9a; mir-927b; mir-375; mir-3720; mir-315; mir-283; bantam; mir-3720; mir-3732; mir-282; mir-71; mir-932 | RNA transport; Calcium signaling pathway |
| 724732 | uncharacterized | mir-275; mir-276; mir-283; mir-927; mir-927b | MAPK signaling pathway; Metabolic pathways; TCA cycle |
| 727507 | uncharacterized | mir-927b; mir-927; mir-375; mir-283; mir-263; mir-14; let-7; mir-1000; mir-316; mir-137; mir-71 | Focal adhesion; ECM-receptor interaction |
| 410013 | sarcoplasmic calcium-binding protein 2（Scp2） | mir-283; mir-3720; mir-87; mir-1; mir-3049; mir-3720; mir-137; mir-379 | Oocyte meiosis; Melanogenesis;Wnt signaling pathway; Olfactory transduction; MAPK signaling pathway; Insulin signaling pathway |
| 724249 | uncharacterized | mir-9a; mir-277; mir-275; mir-263; mir-3783; mir-12; mir-305 | Cell cycle; Progesterone-mediated oocyte maturation |
| 552829 | phospholipase B1, membrane-associated - like | mir-263b; mir-276; mir-927; mir-996; mir-279; mir-996; mir-3783; mir-279b | Vitamin digestion and absorption; Metabolic pathways |
| 725736 | probable oligoribonuclease-like | mir-315; mir-305; mir-137; mir-1000 | Ribosome biogenesis |
| 409278 | apolipoprotein D-like | mir-927b; mir-927; mir-87; mir-3732; mir-7; mir-92a | Melanogenesis; Cell cycle; Wnt signaling pathway; Notch signaling pathway; Jak-STAT signaling pathway |
| 677665 | odorant binding protein 19（Obp19） | bantam;mir-263b;mir-277;mir-283;mir-315;mir-927b;mir-927; mir-9a; mir-12 | Insulin signaling pathway; RNA transpor |
| 726113 | protein-cysteine N-palmitoyltransferase porcupine（por） | mir-315; mir-2944; mir-277; mir-184; mir-100; bantam | Wnt signaling pathway |
| 551379 | chitobiosyldiphosphodolichol beta-mannosyltransferase-like | mir-277; mir-927; mir-927b; mir-305; mir-137; mir-932; mir-3747b; mir-3783; mir-92a | Metabolic pathways |
| 100576436 | uncharacterized | mir-87; mir-375; mir-252; mir-14; mir-125; mir-1; mir-12; mir-305; mir-92a | Spliceosome |
| 409709 | glucocerebrosidase | mir-263; mir-277; mir-927b; mir-3720; mir-281; mir-3720; mir-278; mir-137; mir-379 | Lysosome; Metabolic pathways |
| 551601 | uncharacterized | mir-927b; mir-315; mir-283; mir-252; mir-1000 | RIG-I-like receptor signaling pathway; Tight junction |
| 725376 | uncharacterized | mir-996;mir-929;mir-927b;mir-375;mir-315;mir-283;mir-281;mir-263; mir-279b; mir-7; mir-137; mir-279; mir-996 | Wnt signaling pathway; TGF-beta signaling pathway |
| 725111 | argonaute 3（Ago3） | mir-283; mir-1000; mir-1; mir-3783; mir-12; mir-282; mir-305; mir-137 | Dorso-ventral axis formation |
| 725569 | UDP-xylose and UDP-N-acetylglucosamine transporter-like | mir-2944; mir-283; mir-277; mir-276; mir-275; mir-263b; mir-263; mir-932; mir-316; mir-3747b; mir-305 | Olfactory transduction; Taste transduction |
| 412104 | trafficking protein particle complex subunit 8-like | mir-929; mir-927b; mir-927; mir-2944; mir-277; mir-263; mir-210; mir-2; mir-14; mir-125; mir-137; mir-316; mir-3783; mir-210 | mTOR signaling pathway |
| 406068 | octopamine receptor （Oa1） | mir-1000; mir-3719; mir-3783; mir-3732 |  |
| 678511 | Queen brain-selective protein-1（Qbp-1） | mir-3759; mir-283; mir-252; iab-4; mir-71; mir-1000; mir-3747b; mir-3049 | Bacterial invasion of epithelial cells |
| 413596 | venus kinase receptor （Vkr ） | mir-263b; mir-9a; mir-3720; mir-34; mir-283; mir-275; mir-3049; mir-3720; mir-3783; mir-3732; mir-1; mir-278; mir-282; mir-305; iab-4; mir-137; mir-71; mir-981 | Insulin signaling pathway |
| 100578991 | uncharacterized | mir-9a; mir-929; mir-927b; mir-927; mir-375; mir-283; mir-305; mir-316; mir-3783; mir-12; mir-92a | MAPK signaling pathway |
| 724415 | dynamin-binding protein-like | mir-927b; mir-927; mir-283; mir-277; mir-276; mir-263; mir-100; mir-305; mir-71; mir-1; mir-3049; mir-3783; mir-282 | Regulation of actin cytoskeleton |
| 677664 | odorant binding protein 15 (Obp15) | mir-927b; mir-927; mir-283; mir-277 | ECM-receptor interaction |
| 406101 | odorant binding protein 4（Obp4） | mir-316; mir-3049; mir-12 | p53 signaling pathway |
| 100576439 | coiled-coil domain-containing protein 40-like | mir-9a; mir-929; mir-87; mir-263b; mir-932 | mTOR signaling pathway |
